# Assessment of nutritional value and flavor characteristics of Taihe silky fowl across varieties and marketing ages

**DOI:** 10.1016/j.fochx.2025.103444

**Published:** 2025-12-29

**Authors:** Xiaojun Ju, Ming Zhang, Yunjie Tu, Gaige Ji, Yanju Shan, Yifan Liu, Weidong Zhao, Jingting Shu

**Affiliations:** a Key Laboratory for Poultry Genetics and Breeding of Jiangsu Province, Jiangsu Institute of Poultry Science, Yangzhou 225125, Jiangsu, China.; b Taihe Fengsheng Agriculture & Animal Husbandry Technology Co., Ltd., Ji an 343700, Jiangxi, China

**Keywords:** Chicken, Taihe silky fowl, Nutritional value, Flavor, Multi-methodology

## Abstract

To determine the nutritional value and flavor characteristics of Taihe silky fowl (TH), this study systematically analyzed the differences between amino acids, volatile flavor compounds, and metabolites of Taihe Silky fowl (TH) across different marketing ages (80, 120, 160, and 400 days), as well as among different types of breeds, namely, the yellow-feathered broiler (YF) and the bred silky fowl (ZS), and performed correlation analyses. The results demonstrated that amino acid deposition stabilized after 120 days, while volatile flavor compounds remained abundant at this age. The contents of total amino acids, essential amino acids, nonessential amino acids, umami amino acids, and sweet amino acids in TH were considerably higher than those in YF (*P* ≤ 0.05). In addition, TH contained higher levels of aldehydes, alcohols, ketones, esters, furans, and acids, particularly alcohols, furans, and acids.

Compared with YF, TH exhibited high levels of antioxidants, including diterpenoids and glutathione, while while TH contained higher levels of umami dipeptides, such as β-alanyl-l-lysine, than those in ZS. Considering nutritional value and flavor quality, 120 days were identified as the optimal marketing age for TH. TH exhibited high amino acid content, with richer and more unique flavor profiles, a stronger aroma, and less off-flavor, although it was less suitable for roasting. Succinic acid was a biomarker for superior flavor and nutritional quality in raw chicken meat, and lipid metabolism pathways played a crucial role in flavor development in chicken meat systems. Herein, we conducted a multidimensional analysis of the unique characteristics of TH from both genetic and age perspectives, offering valuable insights into breed improvement and food product development.

## Highlights

• 120-day-old is the best marketing age with stable amino acids and high flavor content.
• Taihe Silky Fowl has higher amino acids, richer volatiles, umami dipeptides & antioxidants.
• Amino acid metabolism causes key breed and age differences.
• Succinic acid may serve as a biomarker for superior flavor and nutritional quality in raw chicken meat.
• Lipid metabolism pathways play a critical role in flavor formation in chicken meat.

## 1 Introduction

Chicken is recognized as one of the most efficient and high-quality sources of animal protein for humans. The high protein and low fat content of chickens have made them highly popular among consumers, and they are highly valued for the nutritional value and palatability they offer. Moreover, chickens with high melanin content are considered a type of meat with high nutritional value, prized texture, and medicinal benefits (Dou et al., 2022). Chinese Taihe silky fowl (TH) is a premium local breed characterized by its snow-white feathers and the presence of melanin in various organs such as skin, meat, and bones (Liao et al., 2022). TH is rich in micronutrients, unsaturated fatty acids, and bioactive compounds, and IT possesses unique medicinal properties (Huang et al., 2024). Its nutritional value and flavor are the main reasons behind consumer acceptance when purchasing and consuming chicken. However, the nutritional value and flavor of TH are highly influenced by breed and age. At present, there is limited in-depth and systematic research on the nutritional value and flavor characteristics of TH.

Different amino acids present in muscle tissue support distinct metabolic functions and variably contribute to nutritional value (Moeller et al., 2010), particularly flavor-related amino acids, which markedly influence the flavor profile and textural properties of poultry meat (Cheng et al., 2024). Flavor is a primary determinant of consumer acceptance when purchasing and consuming meat (Prache et al., 2022). Recently, gas chromatography–ion mobility spectrometry (GC-IMS) has demonstrated its effectiveness in characterizing and analyzing volatile compounds in various food samples (Karpas, 2013). A major advantage of this technique is its ability to detect subtle differences among similar samples (Triba et al., 2015). GC–IMS is with multivariate analysis serves as a convenient and powerful method for characterizing different types of meat (Li et al., 2024; Li et al., 2024). This combined approach enables the characterization and differentiation of livestock and poultry breeds, animals of different ages (Wang et al., 2022), and even muscle sections within the same breed (Duan et al., 2023). To completely exploit the economic potential of silky fowl, recent studies have adopted omics approaches for assessing its characteristics. Metabolomics analyses have been employed to reveal the dynamic changes in the key differential metabolites that affect meat quality, which is influenced by aging (Huang et al., 2024), as well as to uncover differences in nutritional composition between TH and an another similar breed—black-feathered chicken that lays green-shelled eggs (Liao et al., 2022).

On the basis of transcriptomic analyses, candidate genes and pathways potentially involved in melanogenesis have been identified in TH (Huang et al., 2024). Integrated transcriptomic and metabolomic studies have been applied to elucidate the melanogenesis pathway and related meat quality attributes in black-boned chickens (Dou et al., 2022). Using a liquid chromatography–mass spectrometry (LC–MS)-based lipidomics approach, the characteristic lipid composition of TH has been investigated across different ages, sexes, and carcass parts. Variations in the lipid profiles contribute to a better understanding of the pharmacological properties of TH chickens (Mi et al., 2018). Although these studies have notably advanced our understanding of the biological traits of TH, systematic exploration of their nutritional value and flavor attributes from multiple dimensions—such as breed and rearing duration—are scarce.

TH exhibits a range of distinctive characteristics compared with other chicken breeds and at different developmental stages. This study systematically analyzes the differences between amino acids, volatile flavor compounds, and metabolites in the muscle tissues of TH across various marketable ages and in comparison with other chicken breeds. Through a systematic comparison across different marketable ages, the optimal marketing age of TH—when the breed achieves superior nutritional value and flavor quality—is clearly defined, providing precise technical support for practical production. Focusing on TH, a specific rare indigenous breed, this study conducts targeted and systematic comparisons with mainstream commercial chicken breeds, addressing the deficiency of insufficient pertinence in breed comparisons in previous studies. By integrating multidimensional analyses of the nutriome (amino acids), flavorome (volatile flavor compounds detected via GC–IMS), and metabolome, a cohesive investigation spanning nutritional composition, flavor characteristics, and metabolism was realized. This integrative approach not only deepens the understanding of the biological traits of TH but also lays a scientific foundation for breed improvement and product development.

## 2 Materials and methods

### 2.1 Experimental materials

In this experiment, TH of different ages were included, specifically at 80, 120, 160, and 400 days. Thereafter, three different types of broiler chicken breeds were selected: TH (a local dark-boned chicken with silky feathers, 120 days), Yellow-Feathered Broiler (YF, Xinxing Yellow Chicken No. 2, a local breed, 120 days), and Zhisi Chicken (ZS, a selectively bred fast-growing type with silky feathers, 120 days). All the chickens were purchased from Taihe Fengsheng Agriculture and Animal Husbandry Technology Co., Ltd. (China) and were raised in the same batch during the same period. For each breed and age group, 12 chickens with similar body weights were selected from a pool of 500 chickens for experimental measurements. The experimental chickens were electrically stunned and exsanguinated, with all procedures complying with relevant regulations on animal slaughter methods. Bilateral pectoral muscles were immediately collected. One side was minced, homogenized, placed in sealed bags, and stored at −20 °C for amino acid (*n* = 12 per group) and volatile flavor compound analyses (*n* = 9 per group for age groups; *n* = 11 per group for breed groups); both the analyses were completed within 2 days of storage. The other side was sampled from the central portion of the muscle, placed in cryotubes, flash-frozen in liquid nitrogen, and stored at −80 °C for metabolite detection (*n* = 5 per group).

### 2.2 Experimental methods

#### 2.2.1 Determination of amino acids

The amino acid content was determined according to the method described by Li et al. (Li et al., 2023) with minor modifications. Briefly, 0.2 g of meat sample was accurately weighed into a 50 mL hydrolysis tube, to which 20 mL of HCl (6 M) was added. Hydrolysis was performed at 110 °C for 22 h in a forced-air oven. After cooling to room temperature, the hydrolysate was transferred into a 25-mL volumetric flask and diluted to volume with deionized water. Then, 100 μL of the diluted hydrolysate was pipetted into a 15 mL centrifuge tube and evaporated to dryness under a gentle nitrogen stream. Subsequently, 50 μL of derivatization reagent (consisting of ethanol, phenyl isothiocyanate, water, and triethylamine in a volume ratio of 7:1:1:1) was added and the reaction was allowed to proceed at room temperature for 30 min. After derivatization, the mixture was diluted to 0.5 mL with the initial mobile phase, vortexed thoroughly, and filtered through a 0.45 μm organic membrane filter. Finally, the resulting solution was analyzed using an Agilent 1260 high-performance liquid chromatography (HPLC) system.

#### 2.2.2 Determination of volatile flavor compounds

The volatile flavor compounds were analyzed using a FlavourSpec® headspace gas chromatography–ion mobility spectrometry (HS-GC–IMS) system (Model 1H1–00053, G.A.S., Dortmund, Germany) following the reported method (Ju et al., 2025).

Sample preparation and operation procedures: Homogenized chicken breast meat (3.0 g) was weighed into a 20-mL headspace vial. The vial was sealed and incubated at 60 °C for 15 min with agitation at 500 rpm. Subsequently, a 500-μL aliquot of the headspace gas was injected using a syringe heated to 85 °C.

GC analytical conditions: The column used was an FS-SE-54-CB-1 (15 m × 0.53 mm, 1.0 μm). The column temperature was maintained at 60 °C, and the total run time was 20 min. High-purity nitrogen (≥99.999%) was used as the carrier gas. The flow rate was initially set at 2.0 mL/min, held for 2 min, then increased linearly to 10 mL/min over 10 min, followed by a further linear increase to 100 mL/min over 20 min, and finally raised to 150 mL/min over 25 min.

IMS analytical conditions: The drift tube length was 5 cm with a linear voltage of 400 V/cm. The drift tube temperature was set at 45 °C, and high-purity nitrogen (≥99.999%) was used as the drift gas at a flow rate of 150 mL/min. The IMS detector temperature was maintained at 45 °C.

Data analysis: Following data acquisition, the data were processed using the VOCAL software. For visual comparison across samples, a flavor fingerprint plot was first generated using the Gallery plug-in. Subsequently, relative quantification of the volatile compounds was then performed based on the peak volume.

#### 2.2.3 Metabolite analysis and detection

Chicken sample was pretreated according to the method described by Warren et al.(Warren et al., 2017). Briefly, an appropriate amount of sample was accurately weighed into a 2-mL centrifuge tube, followed by the addition of 1000-μL tissue extraction solvent (methanol: chloroform: water = 75:9:16, (*v*/v/v)). The mixture was homogenized using a tissue grinder at 50 Hz for 60 s, and grinding was performed twice. Subsequent processing involved 30 min of ultrasonication at room temperature, followed by 30 min of incubation in an ice bath. After centrifuging at 12,000 rpm for 10 min at 4 °C, the supernatant was collected, concentrated, and dried. The residue was reconstituted in 200 μL of 50% acetonitrile containing 4-ppm 2-amino-3-(2-chlorophenyl)-propionic acid (stored at 4 °C), filtered through a 0.22-μm membrane, and transferred to an injection vial for LC-MS analysis.

Chromatographic separation was performed using a Vanquish UHPLC system (Thermo Fisher Scientific, USA) following the chromatographic conditions established by Zelena et al. (Zelena et al., 2009). Mass spectrometric analysis was conducted on an Orbitrap Exploris 120 mass spectrometer (Thermo Fisher Scientific, USA) with experimental parameters adapted from the methods of Want et al. and Li et al. (Want et al., 2013).

### 2.3 Data analysis and processing

One-way ANOVA was performed using an SPSS 20.0 software (IBM, USA), followed by Tukey's test for post-hoc comparisons. The results were presented as mean ± standard deviation.

## 3 Results and discussion

### 3.1 Comparative study on meat quality of Taihe chickens at different market ages

#### 3.1.1 Comparative study on Amino acid content

**Fig. 1:**
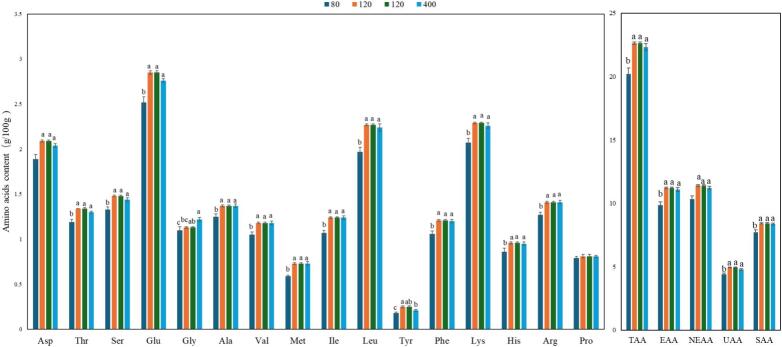
Comparison of amino acid contents in TH at different ages. Aspartic (Asp), Threonine (Thr), Serine (Ser), Glutamic Acid (Glu), Glycine (Gly), Alanine (Ala), Valine (Val), Methionine (Met), Isoleucine (Ile), Leucine (Leu), Tyrosine (Tyr), Phenylalanine (Phe), Lysine (Lys), Histidine (His), Arginine (Arg), and Proline (Pro). Total Amino Acids (TAA), Essential amino acid (EAA) = Thr + Val + Met+Ise + Leu + Phe + Lys + His; Nonessential amino acid (NEAA) = Asp+Ser + Glu + Gly + Ala+Cys + Tyr + Arg + Pro; Umami amino acids (UAA) = Asp+Glu; Sweet amino acids (SAA) = Thr + Ser + Gly + Ala+Lys + Pro.

The results of amino acid content determination and analysis in TH meat at various ages are shown in Fig. 1. Except for glycine and proline, the amino acid content in 80-day-old TH chicken meat was significantly lower than that in 120-, 160-, and 400-day-old chickens (*P* ≤ 0.05). The glycine content in 80-day-old chickens was significantly lower than that in 160 and 400-day-old chickens (*P* ≤ 0.05), while there were no significant differences in proline content among the different age groups (*P* > 0.05). No significant differences were observed in the amino acid content of Taihe chicken meat among the 120-, 160-, and 400-day-old groups (*P* > 0.05). The abovementioned results indicate that age considerably influenced the amino acid content of TH before supplied to market, with younger chickens exhibiting lower amino acid levels. However, beyond 120 days of age, the effect of age on amino acid content diminished. A similar lack of age-related influence on amino acid composition was observed in goat meat (Han et al., 2025). Investigations into ducks of different breeds and ages revealed that amino acid content increased or decreased during the growth phase, while during the later stages of development, the accumulation of nutritional components in duck leg meat could encounter a plateau (Yang et al., 2025). These findings demonstrated that the deposition of amino acids in livestock and poultry meat underwent dynamic changes throughout the growth period and stabilized in the later phases. Considering the nutritional value contributed by amino acids and economic efficiency, 120 days of age was identified as the most suitable slaughter time for TH.

#### 3.1.2 Comparative analysis of volatile flavor compounds

GC-IMS was employed for the rapid and comprehensive analysis of volatile compounds in meat samples from animals of different ages (Wang et al., 2021a). The volatile organic compounds detected via HS-GC–IMS, such as aldehydes, alcohols, ketones, esters, furans, and acids, primarily originated from endogenous enzymatic reactions and the autoxidation of lipids within the postmortem muscle tissue of the chicken. These compounds collectively constituted the fundamental flavor profile of the raw meat. The compounds not only directly defined the inherent aroma characteristics of the fresh ingredient but also were a crucial library of flavor precursors. This library provided the essential substrates for subsequent chemical transformations—such as the Maillard reaction and lipid thermal oxidation—that occur during processing, particularly thermal treatment, thereby profoundly influencing the development of the final flavor in cooked chicken meat.

**Fig. 2:**
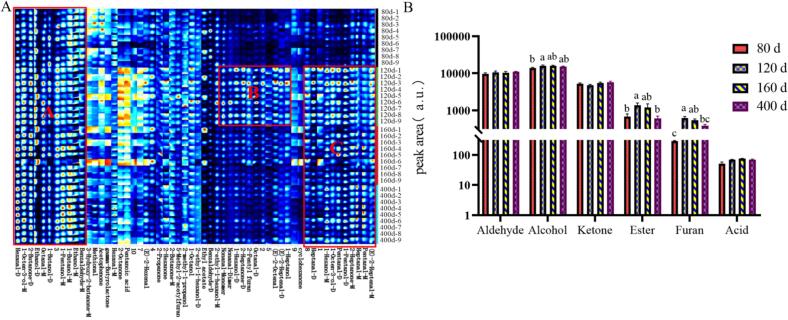
Comparison of volatile flavor compounds in TH at different ages. (A) Fingerprints. (B) Comparative histogram by category.

Characteristic profiles of volatile compounds in TH across various rearing ages revealed distinct compositional features (Fig. 2A). Region A represented common volatile compounds present across all ages, such as hexanal-D, 1-octen-3-ol-M, 2-butanone-D, ethanol-D, octanal-M, 1-butanol-D, 1-pentanol-M, 1-butanol-M, ethanol-M, and benzaldehyde-M. Region B predominantly contained characteristic peaks of 120-day-old chickens, such as nonanal-M, nonanal-D, 1-hexanol-D, 2-heptanone-D, 2-pentylfuran, octanal-D, (E)-2-octenal, (E)-2-heptenal-D, and 1-heptanol. Region C mainly included characteristic peaks found in chickens aged 120–400 days, namely, heptanal-D, 1-octen-3-ol-D, pentanal-D, 1-hexanol-M, 1-pentanol-D, 2-heptanone-M, heptanal-M, pentanal-M, and (E)-2-heptenal-M. The results indicated that the profile of volatile flavor compounds was most abundant at 120 days. Regional analysis showed that the characteristic peaks at 120 days were primarily of aldehydes, such as nonanal (imparting a fatty, citrus-like aroma) (Wang et al., 2024), octanal (with a fruity note) (Wang et al., 2024), and (E)-2-octenal (exhibiting a rosy scent) (Zhao et al., 2025).

As shown in Fig. 2B, the contents of alcohols, esters, furans, and acids in Taihe black-bone chicken meat initially increased and then decreased with the increase in rearing age, reaching their highest levels at 120 days. These values were significantly higher than those at 80 days of age (*P* ≤ 0.05). Alternatively, no significant differences were observed in the contents of aldehydes and ketones (*P* > 0.05). These results suggested that the age-dependent variation in the volatile profile of TH was primarily driven by alcohols, esters, furans, and acids. Of these, alcohols constituted the largest proportion and played a crucial role in shaping the olfactory perception of chicken meat by imparting pleasant floral and fruity aromas (Calkins et al., 2007). Unsaturated alcohols possessed low odor thresholds, making them important contributors to flavor (Ju et al., 2025). Esters, formed from reactions between alcohols and acids, provided a wide range of fruity or creamy notes (Forney et al., 2017) and could be used as additives for enhancing desirable flavors in meat products (Dong et al., 2024). Moreover, furans and related heterocyclic compounds, primarily generated via the Maillard reaction (Ba et al., 2013), add further complexity. Overall, these findings indicated that the flavor profile of TH was the most complex and rich at 120 days, consistent with the peak contents of key volatile compounds (alcohols, esters, furans, and acids) observed at this rearing age.

The contents of (E)-2-hexenal, hexanal, pentanal, 1-hexanol, and 2-butanone progressively increased, reaching their highest levels at 400 days of age. Alternatively, the contents of 1-hexanol and pentanoic acid initially increased and then decreased, peaking at 160 days. Many compounds, such as (E)-2-octenal, heptanal, nonanal, octanal, 1-octen-3-ol, ethanol, 2-heptanone, cyclohexanone, ethyl acetate, 5-methyl-2-acetylfuran, and 2-pentylfuran, demonstrated an initial increase followed by a decrease, with their highest concentrations observed at 120 days. Conversely, the contents of benzaldehyde, methional, 1-butanol, 2-ethyl-1-hexanol, 2-propanone, acetophenone, and γ-butyrolactone demonstrated either a decreasing or a fluctuating (decreasing then increasing) trend, showing their highest values at 80 days (Supplementary Table 1).

#### 3.1.3 Comparative analysis of differential metabolites

Metabolites with VIP > 1 and *p* < 0.05, identified across different ages, were considered significantly differential. Seventy-one differential metabolites were screened.

**Fig. 3:**
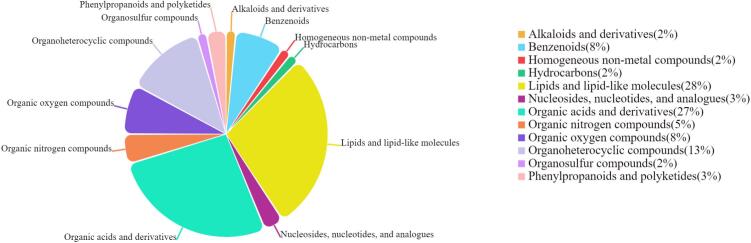
Classification and proportion of metabolites in meat of TH at different ages.

Classification analysis of age-related differential metabolites revealed that lipids and lipid-like molecules (28%) as well as organic acids and their derivatives (27%) shared the maximum proportion (Fig. 3), indicating that lipid metabolism and organic acid balance were the key regulatory pathways involved in the changes observed in TH chicken meat at different ages. Among lipid molecules, the contents of various phosphatidylcholines (e.g., PC(O-16:0/20:0)) and lysophospholipids (e.g., LysoPC(0,0/18,0)) considerably changed with age.The metabolism of intramuscular phospholipids imparted fresh meat with a unique texture and appearance (e.g., marbling) and affected the texture, flavor, nutritional value, and other quality attributes of meat and meat products (Li et al., 2020; Zhang et al., 2023; Zou et al., 2023). Concurrently, changes in molecules such as glycerol 3-phosphate (a core precursor for lipid synthesis) and linolenic acid (a key polyunsaturated fatty acid) further corroborated the active role of the lipid metabolic network, laying the foundation for the accumulation of flavor precursors at specific ages. Among organic acids and derivatives, succinic acid and L-isoleucine exhibited pronounced age-dependent trends. Succinic acid peaked at D120, indicating that the muscle energy metabolism was the most vigorous at this stage, and its role as a flavor precursor could influence the umami taste and tenderness of the meat (Mills et al., 2018; Zappaterra et al., 2016). L-Isoleucine was involved in protein synthesis (Nie et al., 2018) and could also improve the meat quality by promoting myocyte differentiation and intramyocellular fat accumulation (Liu et al., 2021). The highest level of L-isoleucine was observed at D80, likely owing to active protein deposition in the early growth stage; its dynamic changes reflected the characteristics of muscle growth and amino acid metabolism. The variations in these two categories of metabolites illustrated the synergistic role of energy metabolism and protein metabolism in meat quality formation, providing a basis for targeted nutritional regulation.

**Fig. 4:**
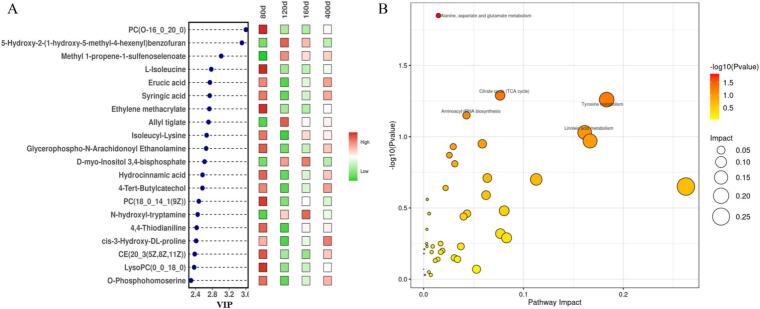
Differential metabolites and metabolic pathways in TH at different days of age. (A) Lollipop heatmap of top 20 differential metabolites; (B) Enriched pathways of differential metabolites.

Fig. 4A shows the top 20 differential metabolites among TH at different ages using a lollipop heatmap. With VIP as the horizontal axis, the VIP values of these differential metabolites were all above 2.4, particularly with PC(O-16:0/20:0) exhibiting the maximum significant difference. As discussed previously, PC(O-16:0_20:0) was present at relatively low concentrations in TH and showed a strong negative correlation with polyunsaturated fatty acids and aldehydes—key compounds that contribute to meat flavor. The abundance of PC(O-16:0_20:0) demonstrated an initial decrease followed by an increase, peaking at 80 days and reaching its lowest level at 120 days of age.

Hydroxycinnamic acid, a phenolic acid and a major metabolite of phenylalanine metabolism, exhibited a sweet balsamic aroma and was the precursor of flavor compounds (Xu et al., 2022). Hydroxycinnamic acid content peaked at 80 days, suggesting that it was the key component that contributed to the “characteristic flavor” of poultry meat from younger birds and played a crucial role in the flavor formation of young broilers. Erucic acid has been reported to stimulate fat accumulation in skeletal muscle and increase protein levels (Yu et al., 2025); however, studies ave. demonstrated that it could exhibit implications for human health (Patra et al., 2025). Erucic acid reached its highest level at 80 days of age. Similarly, Yu et al. also observed higher erucic acid content in young geese than in adult ones (Yu et al., 2020). The functional value of erucic acid in chicken meat requires further investigation. Many differentially abundant metabolites were identified at 80 days, and notable differences in metabolite profiles were observed between 80 and 120 days. Nevertheless, the specific roles of these metabolites in meat nutrition or flavor remained unclear, which could partly explain the unique meat quality attributes of younger broilers.

The main metabolic pathways are shown in Fig. 4B, with five considerably different metabolic routes—valine, leucine and isoleucine biosynthesis; alanine, aspartate and glutamate metabolism; lysine degradation, oxidative phosphorylation; and D-amino acid metabolism. These metabolic pathways were primarily associated with amino acid metabolism and validated the crucial role of amino acids in the nutritional and flavor profiles of meat. These amino acids not only served as the fundamental units for protein synthesis but also participated in various metabolic processes, such as transamination and decarboxylation, leading to the formation of volatile flavor compounds, such as aldehydes, ketones, and amines, which considerably influence meat flavor (Chen et al., 2024). Similarly, Han et al. demonstrated a strong correlation between age and amino acid metabolism in their metabolomic analysis of goats from different age groups (Han et al., 2025).

### 3.2 Comparative study on meat quality between TH, YF and ZS

#### 3.2.1 Comparative study on amino acid content

**Fig. 5:**
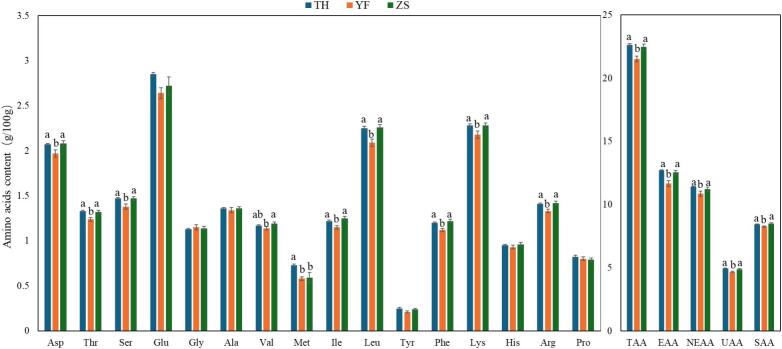
Comparison of amino acid contents between TH, YF and ZS.

The results of amino acid content determination and analysis in TH, YF, and ZS are shown in Fig. 5. The contents of aspartic acid, threonine, serine, isoleucine, leucine, tyrosine, phenylalanine, lysine, arginine, total amino acids (TAAs), essential amino acids (EAAs), nonessential amino acids (NEAAs), umami amino acids (UAAs), and sweet amino acids (SAAs) in TH were significantly higher than those in YF (*P* ≤ 0.05), but did not differ significantly from those in ZS (*P* > 0.05). The methionine content was significantly higher in TH than in both YF and TH (*P* ≤ 0.05). No significant difference was observed in valine content between TH, YF, and ZS (*P* > 0.05).

Amino acids are the essential components of proteins, and the types of amino acids present in chicken meat are associated with its nutritional value and metabolic functions (Zhang et al., 2023). This study observed that both varieties of black-boned chickens contained higher levels of amino acids, suggesting that their nutritional and functional values could be superior to those of YFs. The content of amino acids was observed to be closely related to meat flavor (Li et al., 2022). Maillard reactions and Strecker degradation of amino acids considerably influenced the volatile flavor compounds in meat (Chen et al., 2024; Li et al., 2021). Most amino acids involved in these reactions—such as lysine, arginine, phenylalanine, leucine, isoleucine, tyrosine, and methionine—were markedly higher in black-boned chickens than in YFs. The study by Yang et al. (Yang et al., 2025) also reported that the contents of EAAs, NEAAs, and flavor-related amino acids were higher in black-boned chickens those in Arbor Acres (AA) broilers, indicating that the black-boned chickens possessed superior flavor characteristics.

#### 3.2.2 Comparative analysis of volatile flavor compounds

GC–IMS has emerged as an approach for detecting trace amounts of VOCs (Su et al., 2025). To investigate and discriminate flavor compounds in Taihe silky chicken, this study employed GC–IMS for characterizing meats of three different types of chicken.

**Fig. 6:**
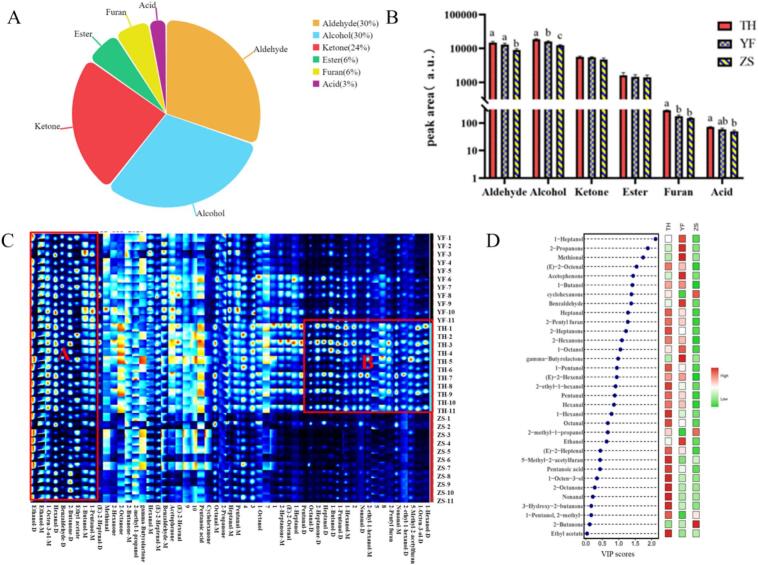
Comparison of volatile flavor compounds among TH, ZS and YF. (A) Categorical pie chart. (B) Comparative histogram by category. (C) Fingerprints. YF1–11: samples from 11 Yellow-feather broilers. TH1–11: samples from 11 Taihe silky fowl chickens. ZS1–11: samples from 11 Zhisi chickens. (D) Dot-bar heat map. (For interpretation of the references to colour in this figure legend, the reader is referred to the web version of this article.)

In chicken meat, 33 volatile flavor compounds were detected, predominated by aldehydes, alcohols, and ketones (Fig. 6A). A comparative analysis revealed that the aldehyde content in TH was significantly higher than that in ZS (*P* ≤ 0.05), while the contents of alcohols and furans were significantly high compared with YF and ZS (*P* ≤ 0.05) (Fig. 6B). Upon analyzing, it was revealed that the flavor compounds of TH markedly differed from those of YF and ZS. The contents of aldehyde, alcohol, ketone, ester, furan, and acid classes were all relatively high, particularly alcohol, furan, and acid. The increased levels of the key flavor-active compounds in TH indicated a more complex and abundant flavor profile. As established, alcohols contributed by imparting pleasant floral and fruity notes and were crucial for aroma perception. Furans, primarily products of the Maillard reaction and lipid degradation, imparted sweet, caramel, and nutty aromas, enriching the flavor complexity. Although many acids exhibit high detection thresholds. They could contribute to overall flavor perception. Herein, a short-chain fatty acid—pentanoic acid—was used. Song et al. reported that short-chain fatty acids with low odor thresholds could contribute to unique flavor characteristics (Song et al., 2011), potentially lending a distinctive cheesy or sweaty note that adds to the flavor diversity. The concurrent increase in alcohols and acids provided a substrate pool for ester formation, which could generate additional fruity esters postprocessing or during cooking, further enhancing the flavor. Therefore, the synergistic increase of these compound classes not only differentiated TH from commercial breeds but also underpinned its richer and more layered flavor profile.

The characteristic profiling of volatile compounds in chicken meat revealed distinct patterns among the three chicken varieties (Fig. 6C), Region A contained common volatiles shared by all varieties, such as 1-pentanol-M, 1-butanol-M, ethyl acetate, 2-butanone-D, benzaldehyde-D, hexanal-D, 1-octen-3-ol-M, ethanol-M and ethanol-D. Region B exhibited characteristic markers that were unique to TH, featuring 1-hexanol-D, 1-octen-3-ol-D, 5-methyl-2-acetylfuran, 2-ethyl-1-hexanol-D, nonanal-M, 2-pentyl furan, 2-ethyl-1-hexanol-M, nonanal-D, 1-hexanol-M, 1-pentanol-D, 1-butanol-D, heptanal-D, 2-heptanone-D and octanal-D. The flavor of TH was richer, more unique, and more intense.

The contents of (E)-2-heptenal, (E)-2-octenal, nonanal, octanal, 1-hexanol, 1-octen-3-ol, 2-ethyl-1-hexanol, 2-heptanone, 2-octanone, 3-hydroxy-2-butanone, 5-methyl-2-acetylfuran, and pentanoic acid in TH were considerably higher than those in both YF and ZS (*P* ≤ 0.05). The contents of (E)-2-hexenal, benzaldehyde, hexanal, pentanal, heptanal, 1-butanol, 1-heptanol, 1-pentanol, 1-octanol, 2-propanone, 2-hexanone, and 2-pentylfuran in TH were significantly higher than those in ZS (*P* ≤ 0.05). The content of 2-methyl-1-propanol in TH was significantly higher than that in YF (*P* ≤ 0.05), whereas the contents of benzaldehyde, methional, 1-heptanol, ethanol, acetophenone, and gamma-butyrolactone were significantly lower than those in YF (*P* ≤ 0.05) (Table S1).

Nonanal and octanal, which were oxidation products of linoleic acid (Mingwu et al., 2016), contributed distinct aroma profiles: Octanal imparted a fruity note, while nonanal exhibited a fragrant odor (Wang et al., 2024). Hexanol was derived from the reduction of hexanal and exhibited a pungent, intense vanilla-like odor (Zhao et al., 2025). 1-Octen-3-ol, identified as one of the key alcohols in chicken meat, was generated by oxidizing linoleic acid, and it contributed most significantly to the overall flavor profile of Chinese native chicken breeds (Jin et al., 2021). 1-Octen-3-ol was characterized by an extremely low sensory threshold and a distinct mushroom-like aroma (Wang et al., 2022). Previous studies have shown a higher relative content of 1-octen-3-ol in the breast muscle of black-boned chickens than of AA broilers (Yang et al., 2025). Ketones, as products of lipid oxidation, typically imparted fruity, milky, and creamy notes. 2-Heptanone was known for its buttery, cheese-like aroma (Purriños et al., 2012), while 3-hydroxy-2-butanone endowed the meat with a pleasant, milky flavor (Li et al., 2022; Wang et al., 2024). The contents of these compounds in TH were higher than those in ZS and YF. Furthermore, benzaldehyde, which could potentially negatively impact the olfactory profile of the meat owing to its almond and caramel-like odor (Wang et al., 2024), was detected in all chicken samples and was more abundant in YFs. This suggested that the meat of local black-boned chicken contained a high level of pleasant flavor compounds.

As shown in the heatmap of Fig. 6D, the flavor compounds that most significantly influenced the different varieties were 1-heptanol, 2-propanone, methional, and (E)-2-octenal. 1-heptanol, which imparted a floral aroma, was identified as the key volatile compound in roasted chicken (Wang et al., 2023). The content of 1-heptanol was the highest in YF chicken and relatively low in silky fowl, suggesting that silky fowl was not suitable for roasting. 2-Propanone, a lipid degradation product, was perceived as a pungent odor (Kerth et al., 2015) and was reported to exhibit a low detection threshold associated with bloody and liver-like aftertastes (Insausti et al., 2021). 2-Propanone content was the highest in YF chicken, lower in silky fowl, and lowest in ZS chicken, indicating that silky fowl—especially the ZS breed—likely exhibited a milder meaty odor. Methional was produced through a series of complex chemical reactions during heating (e.g., roasting, frying, or boiling) that degraded methionine; hence, its content in raw meat was extremely low. (E)-2-octenal, as discussed previously, imparted a rose-like aroma and was the most abundant in TH chicken. The increased abundance of (E)-2-octenal indicated that compared with YF chickens, the raw meat of silky fowl exhibited a different foundation in flavor compound composition. Specifically, the raw meat of silky fowl contained lower contents of 1-heptanol (a key flavor compound in roasted chicken) and 2-propanone, suggesting that it could develop a distinct flavor profile compared with YF chickens after thermal processing (e.g., roasting). This finding, based on raw meat data, warrants future validation through systematic cooking experiments (e.g., roasting at different temperatures and durations) to clarify the thermal processing characteristics of silky fowl meat.

#### 3.2.3 Comparative analysis of differential metabolites

**Fig. 7:**
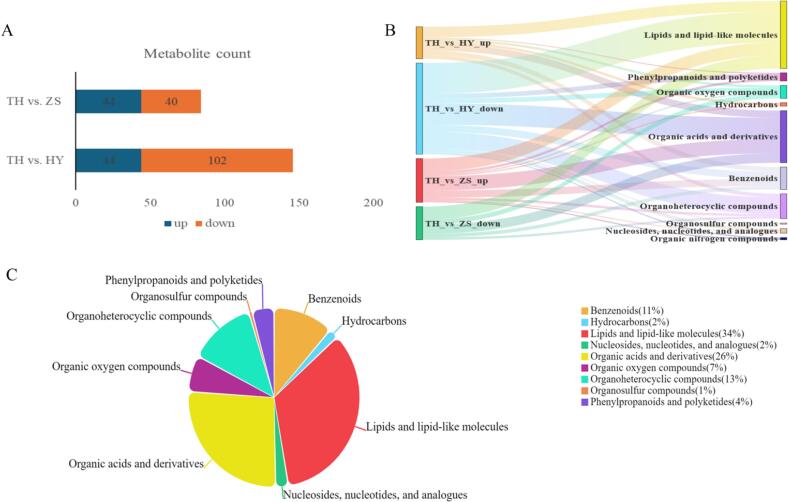
Metabolite analysis among different breeds. (A) Number of differential metabolites. (B/C) Classification information of differential metabolites. TH_vs_HY_up/ TH_vs_HY_down: the metabolites significantly more/ less abundant in TH than HY; TH_vs_ZS_up/ TH_vs_ZS_down: the metabolites significantly more/ less abundant in TH than ZS.

HPLC-MS/MS enabled the discrimination and identification of different species and poultry breeds (Häfner et al., 2021; Zhang et al., 2022). Metabolites with VIP > 1, and *P* < 0.05, identified between different groups, were considered significantly differential. There were 147 significantly differential metabolites between TH and HY, and 85 significantly differential metabolites between TH and ZS (Fig. 7A).

The classification information of the significantly differential metabolites is shown in Fig. 7B and C. The metabolites with significant differences between TH and HY were mainly lipids and lipid-like molecules, organic acids and derivatives, organoheterocyclic compounds, and benzenoids. The metabolites with significant differences between TH and ZS were mainly organic acids and derivatives, lipids and lipid-like molecules, and benzenoids. Lipids and lipid-like molecules as well as organic acids and their derivatives remained the most significant categories, further underscoring their crucial roles in chicken meat.

**Fig. 8:**
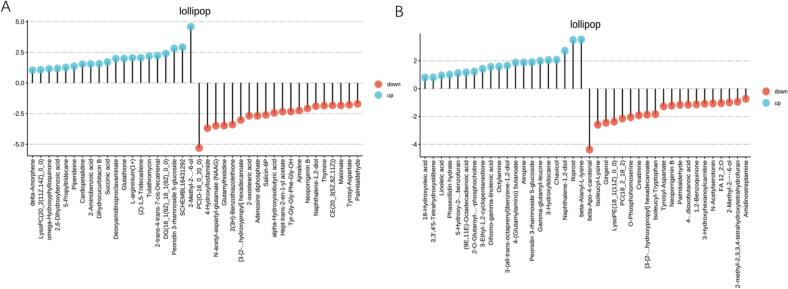
The top 20 up- and downregulated metabolites. The lollipop diagram showing the top 20 differential metabolites between the TH vs HY group (A) and TH vs ZS group (B).

As shown in Fig. 8A, the substances that exhibited the greatest differences between TH and HY were 2-Methyl-2-(4,8,12-trimethyltridecyl)-3,4,4a,5,6,7,8,8a-octahydrochromen-6-ol and PC(O-16:0/20:0). 2-methyl-2-(4,8,12-trimethyltridecyl)-3,4,4a,5,6,7,8,8a-octahydrochromen-6-ol was identified as the metabolite exhibiting the most significant difference between TH and YF. This metabolite predominated the silky fowl and was classified as a diterpenoid. Diterpenoids were known for their antioxidant and antimicrobial properties, as well as their ability to enhance flavor profiles, indicating that they had broad application potential (Jinxi et al., 2024). PC(O-16_0_20_0), a member of the glycerol phospholipid family, was an essential component of biological membranes (Dean et al., 2018). PC(O-16_0_20_0) exhibited a strong negative correlation with polyunsaturated fatty acids and aldehydes, which were recognized as crucial flavor compounds in meat (Feng et al., 2022; Wang et al., 2022). Moreover, the presence of C20:0 (arachidic acid), a saturated fatty acid esterified at the sn − 2 position, was considered potentially burdensome to lipid metabolism when consumed in excess. The content of PC(O-16:0_20:0) was found to be significantly lower in TH than in YF. Glutathione, upregulated in TH chickens, is a key antioxidant and a flavor substance in meat (Setyabrata et al., 2021). Glutathione contributed to a distinctive umami richness characterized by continuity, mouthfulness, and thickness—particularly in chicken broth. When added to aqueous solutions containing sucrose, NaCl, or monosodium glutamate, glutathione was shown to selectively enhance the perception of sweetness, saltiness, and umami (Ohsu et al., 2010). This was thought to partly explain the reason behind TH being traditionally valued for their beauty-nourishing properties and suitability for preparing soups.

As shown in Fig. 8B, β-alanyl-l-lysine a dipeptide formed by a peptide bond between β-alanyl-l-lysine, was identified as a metabolite that exhibited marked differences between TH and ZS. Such peptides this were observed to participate in the Maillard reaction and Strecker degradation, generating hydrogen sulfide, ammonia, cysteamine, thiazoles, thiophenes, and their derivatives, all of which were reported to contribute to meat flavor (Huang et al., 2022; Mottram, 1994; Que et al., 2023). β-alanyl-l-lysine could be hydrolyzed into its two constituent free amino acids—β-alanine and l-lysine. Among these, l-lysine was reported to influence meat texture and water-holding capacity (Zhang et al., 2020). When β-alanine was combined with l-histidine, it formed carnosine, a flavor precursor peptide (Blancquaert et al., 2016). This histidine-containing umami dipeptide was abundant in livestock and poultry, it considerably contributed to the umami taste in meat (Kim et al., 2024). The content of β-alanyl-l-lysine was markedly higher in TH than in ZS. Furthermore, the creatinine content in TH was notably lower than that in ZS. Creatinine has been identified as one of the precursors contributing to the formation of heterocyclic amines (HAs) (Yang et al., 2019), carcinogenic and mutagenic compounds generated during the thermal processing of meat products (Chen et al., 2020). The reduction in creatinine content was observed to effectively suppress the formation of HAs, thereby enhancing food safety and offering more benefits for human health (Deng et al., 2025). Overall, these findings demonstrated that TH exhibited superior edible quality and nutritional value compared with YF and ZS.

**Fig. 9:**
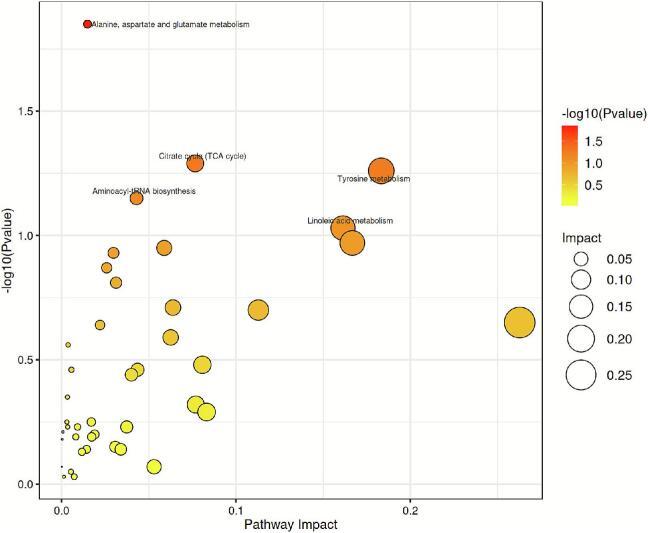
Enriched pathways of differential metabolites.

The main metabolic pathways are shown in Fig. 9, Alanine, aspartate and glutamate metabolism was the only important pathway.

In a study on breed-specific characteristics and hybridization effects in cattle, Zhao et al. (Zhao et al., 2025) reported that differentially abundant metabolites were primarily associated with amino acid metabolic pathways. Similarly, while investigating metabolic differences between TH and another comparable breed, black-feathered chicken, Liao et al. (Liao et al., 2022) observed marked differences in protein synthesis and amino acid transport in TH compared with those in black-feathered chickens. The alanine, aspartate, and glutamate metabolism pathway was the major differential metabolic pathway between TH and other silky fowls. The alanine, aspartate, and glutamate metabolism pathway played a central role in the synthesis and regulation of umami compounds during postmortem quality formation across various livestock and poultry species, demonstrating a universal function that remained conserved cross-species (Duan et al., 2023; Zhao et al., 2022). This pathway was the core hub within the postmortem muscle metabolic network, regulating the generation and transformation of key UAAs—such as glutamate and aspartate (Ge et al., 2022; Wang et al., 2022). The pathway constituted the metabolic foundation for flavor precursor substances. During processing, This pathway was dynamically activated via protein hydrolysis, driving the accumulation of umami compounds and the final development of flavor (Fu et al., 2022). Consequently, this pathway likely acted as the metabolic hub underlying the unique meat quality characteristics of the TH among black-boned chickens.

### 3.3 Correlation analysis of amino acid composition, flavor compound changes, and differential metabolites

**Fig. 10:**
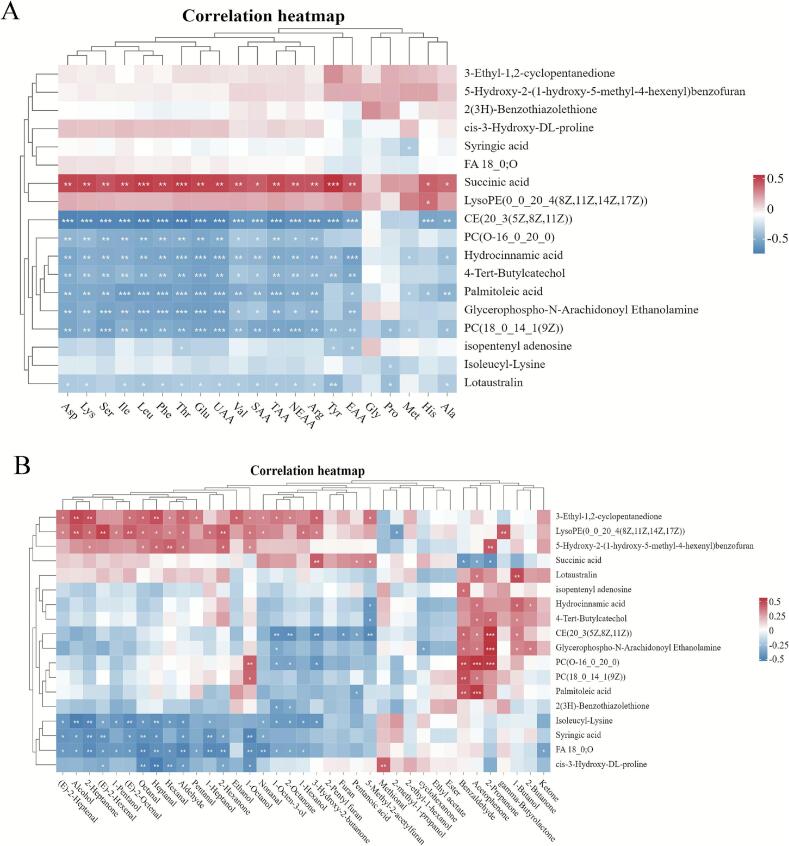
Correlation analysis of amino acid composition, flavor compound changes, and differential metabolites. (A) Heatmap showing the correlation between amino acids and differential metabolites; (B) Heatmap showing the correlation between volatile flavor compounds and differential metabolites.

To investigate the association between differential metabolites and amino acids, a Pearson correlation analysis was performed between the differential metabolites jointly screened in 3.1.3, 3.2.3 and the amino acid composition and a heatmap was generated (Fig. 10). The results in Fig. 10A demonstrate that metabolites such as succinic acid exhibited a significant positive correlation (*P* < 0.05) with most amino acids, particularly UAAs and SAAs. By contrast, CE(20:3(5Z,8Z,11Z)), PC(O-16:0/20:0), hydrocinnamic acid, 4-tert-butylcatechol, palmitoleic acid, glycerophospho-N-arachidonoyl ethanolamine, PC(18:0/14:1(9Z)), and lotaustralin exhibited a significant negative correlation (*P* < 0.05) with most amino acids. The results in Fig. 10B indicate that CE(20:3(5Z,8Z,11Z)), glycerophospho-N-arachidonoyl ethanolamine, and PC(O-16:0/20:0) were significantly positively correlated (*P* < 0.01) with 2-propanone. Furthermore, PC(O-16:0/20:0) and palmitoleic acid exhibited a highly significant positive correlation (*P* < 0.01) with acetophenone.

Succinic acid exhibited a significant positive correlation with the UAA/SAA pool. This finding aligns with the observations of Zhang et al., indicating that succinic acid is not only an energy source (within the TCA cycle) and a precursor for umami compounds but also a direct regulator of muscle fiber type and promoter of intramuscular fat deposition. Consequently, in chicken meat, a higher level of succinic acid likely drove efficient oxidative metabolism, providing an energetic foundation for Type I/IIa slow-twitch muscle fibers (which could enhance meat tenderness and flavor) while supplying carbon skeletons for the accumulation of amino acids (Zhang et al., 2022). This suggests that succinic acid can be a potential biomarker that indicates superior flavor and nutritional quality in raw chicken meat and that further research is required to experimentally validate this association.

Several lipids and antioxidant phenols exhibited negative correlations with most amino acids while showing significant positive correlations with the volatile flavor compounds—2-propanone and acetophenone. To our knowledge, this specific correlation pattern between lipids/antioxidant phenols and amino acids has not been directly reported in previous literatures. However, amino acid and lipid metabolisms are not independent processes; they are closely interconnected in terms of material and energy flow. The catabolism of amino acids could directly provide carbon skeleton precursors for lipid synthesis while being alternative substrates for mitochondrial respiration to supply energy for such synthesis (Cai et al., 2022). Moreover, certain amino acid metabolic pathways could regulate the synthesis of key lipids such as phosphatidylcholine (Blachier et al., 2020). Based on the mechanisms discussed above, we propose that the “negative correlation” can be attributed to the distinct functional roles of the corresponding compounds. Volatile ketones (e.g., 2-propanone and acetophenone) were primarily derived from lipid degradation (Wang et al., 2021b), a process that involved the oxidative degradation of free fatty acids, amino acid oxidation, and interactions between proteins and volatile compounds. (Xin et al., 2025). Therefore, the strong correlations among lipid metabolites, volatile flavor compounds, and amino acids highlighted the crucial role of lipid metabolism pathways in flavor formation in the chicken meat system studied here.

## 4 Conclusion

This study systematically analyzed the meat quality characteristics of TH. Analysis of age indicated that 120 days was the optimal marketing age for TH, at which point volatile flavor compounds were the most abundant and amino acid deposition was stabilized. Amino acid metabolism was identified as the core pathway affected by age. Comparative breed analysis revealed that, compared with YF, TH exhibited a comprehensive nutritional advantage, with markedly higher contents of TAAs, EAAs, UAAs, and SAAs (*P* ≤ 0.05). Regarding flavor compounds, TH exhibited higher levels of aldehydes, alcohols, ketones, esters, furans, and acids—particularly alcohols, furans, and acids—providing the material basis for its stronger and more distinctive flavor and aroma. At metabolic level, TH contained higher levels of antioxidants (e.g., diterpenoids and glutathione) and umami dipeptides. Compared with selectively bred silky fowl (ZS), TH demonstrated a more pronounced umami character. Furthermore, alanine, aspartate, and glutamate metabolism were identified as the key differential metabolic pathways distinguishing TH from other silky fowl varieties. Succinic acid was a potential biomarker for superior flavor and nutritional quality in raw chicken meat. Lipid metabolism pathways were observed to play a crucial role in flavor formation in chicken meat systems. This study provides important parameters for production practices, and the identified characteristic flavor compounds and metabolic targets establish a theoretical foundation for improving chicken meat quality through breeding or nutritional strategies. Future researchers should validate the benefits of this optimal age under different rearing systems and further elucidate the molecular mechanisms underlying flavor formation to better guide the product development.

## CRediT authorship contribution statement

**Xiaojun Ju:** Writing – original draft, Data curation, Conceptualization. **Ming Zhang:** Formal analysis, Data curation. **Yunjie Tu:** Validation, Methodology. **Gaige Ji:** Resources, Investigation. **Yanju Shan:** Writing – review & editing, Resources, Data curation. **Yifan Liu:** Project administration, Methodology. **Weidong Zhao:** Supervision, Resources, Conceptualization. **Jingting Shu:** Writing – review & editing, Project administration, Funding acquisition.

## Ethics statement

All animal experiments were performed in accordance with the protocol of the Animal Use Committee of the Chinese Ministry of Agriculture and were approved by the Animal Care and Use Committee at the Jiangsu Institute of Poultry Science (Approval ID: S20240426, Yangzhou, China). All experiments were performed in accordance with relevant guidelines and regulations. The animals were euthanized according to the American Veterinary Medical Association (AVMA) Guidelines for the Euthanasia of Animals (2020). All efforts were made to minimize animal suffering. The reporting in the manuscript follows the recommendations in the ARRIVE guidelines, and were in accordance with relevant guidelines and regulations.

## Declaration of competing interest

The authors declare that they have no known competing financial interests or personal relationships that could have appeared to influence the work reported in this paper.

## Data Availability

No data was used for the research described in the article.

## Appendix A Supplementary data

Supplementary material 1 In the same row, values with no letter or the same letters mean no significant difference (*P*>0.05), while with different small letters mean significant difference (*P*<0.05). Supplementary material 1
